# Epigenome-wide association study of dietary fatty acid intake

**DOI:** 10.1186/s13148-024-01643-9

**Published:** 2024-02-16

**Authors:** Julia Lange de Luna, Aayah Nounu, Sonja Neumeyer, Lucy Sinke, Rory Wilson, Fabian Hellbach, Pamela R. Matías-García, Thomas Delerue, Juliane Winkelmann, Annette Peters, Barbara Thorand, Marian Beekman, Bastiaan T. Heijmans, Eline Slagboom, Christian Gieger, Jakob Linseisen, Melanie Waldenberger

**Affiliations:** 1 Research Unit Molecular Epidemiology, Institute of Epidemiology, Helmholtz Zentrum Munich, German Research Center for Environmental Health, Ingolstädter Landstraße 1, 85764 Neuherberg, Germany; 2 Institute of Epidemiology, Helmholtz Zentrum Munich, German Research Center for Environmental Health, 85764 Neuherberg, Germany; 3 Molecular Epidemiology, Department of Biomedical Data Science, Leiden University Medical Center, 2333 ZC Leiden, The Netherlands; 4 Epidemiology, Faculty of Medicine, University of Augsburg, University Hospital of Augsburg, 86156 Augsburg, Germany; 5 Institute for Medical Information Processing, Biometry and Epidemiology-IBE, LMU Munich, 80539 Munich, Germany; 6 Institute of Neurogenomics, Helmholtz Zentrum Munich, German Research Center for Environmental Health, 85764 Neuherberg, Germany; 7 Klinikum Rechts Der Isar, Chair Neurogenetics, Technical University of Munich, Munich, Germany; 8 Klinikum Rechts Der Isar, Institute of Human Genetics, Technical University of Munich, Munich, Germany

**Keywords:** DNA methylation, EWAS, PUFA n-3, PUFA n-6, Fatty acids, Docosapentaenoic acid, Stearidonic acid, Eicosapentaenoic acid, Eicosadienoic acid

## Abstract

**Background:**

Dietary intake of n-3 polyunsaturated fatty acids (PUFA) may have a protective effect on the development of cardiovascular diseases, diabetes, depression and cancer, while a high intake of n-6 PUFA was often reported to be associated with inflammation-related traits. The effect of PUFAs on health outcomes might be mediated by DNA methylation (DNAm). The aim of our study is to identify the impact of PUFA intake on DNAm in the Cooperative Health Research in the Region of Augsburg (KORA) FF4 cohort and the Leiden Longevity Study (LLS).

**Results:**

DNA methylation levels were measured in whole blood from the population-based KORA FF4 study (*N* = 1354) and LLS (*N* = 448), using the Illumina MethylationEPIC BeadChip and Illumina HumanMethylation450 array, respectively. We assessed associations between DNAm and intake of eight and four PUFAs in KORA and LLS, respectively. Where possible, results were meta-analyzed.

Below the Bonferroni correction threshold (*p* < 7.17 × 10^–8^), we identified two differentially methylated positions (DMPs) associated with PUFA intake in the KORA study. The DMP cg19937480, annotated to gene *PRDX1*, was positively associated with docosahexaenoic acid (DHA) in model 1 (beta: 2.00 × 10^–5^, 95%CI: 1.28 × 10^–5^-2.73 × 10^–5^, *P* value: 6.98 × 10^–8^), while cg05041783, annotated to gene *MARK2*, was positively associated with docosapentaenoic acid (DPA) in our fully adjusted model (beta: 9.80 × 10^–5^, 95%CI: 6.25 × 10^–5^-1.33 × 10^–4^, *P* value: 6.75 × 10^–8^). In the meta-analysis, we identified the CpG site (cg15951061), annotated to gene *CDCA7L* below Bonferroni correction (1.23 × 10^–7^) associated with eicosapentaenoic acid (EPA) intake in model 1 (beta: 2.00 × 10^–5^, 95% CI: 1.27 × 10^–5^–2.73 × 10^–5^, *P* value = 5.99 × 10^–8^) and we confirmed the association of cg19937480 with DHA in both models 1 and 2 (beta: 2.07 × 10^–5^, 95% CI: 1.31 × 10^–5^–2.83 × 10^–5^, *P* value = 1.00 × 10^–7^ and beta: 2.19 × 10^–5^, 95% CI: 1.41 × 10^–5^–2.97 × 10^–5^, *P* value = 5.91 × 10^–8^ respectively).

**Conclusions:**

Our study identified three CpG sites associated with PUFA intake. The mechanisms of these sites remain largely unexplored, highlighting the novelty of our findings. Further research is essential to understand the links between CpG site methylation and PUFA outcomes.

**Supplementary Information:**

The online version contains supplementary material available at 10.1186/s13148-024-01643-9.

## Introduction

Polyunsaturated fatty acids (PUFAs) are a group of fatty acids that contain multiple double bonds between carbon atoms and can be classified into n-3 or n-6 depending on the position of the first double bond from the methyl terminal [1]. They are of great importance in the western diet [2] and studies showed that the consumption of n-3 PUFA has a protective effect on the development of chronic diseases, such as cardiovascular disease [3], diabetes [4], depression [5] and cancer [6, 7]. They can be found in high amounts in seeds of chia, perilla and flax as well as in fish and fish oil [8]. n-6 PUFA consumption is associated with the synthesis of proinflammatory eicosanoids and with inflammation-related pathophysiologic events or diseases, such as nonalcoholic fatty liver disease, cardiovascular disease, inflammatory bowel disease, rheumatoid arthritis and Alzheimer’s disease [9]. These PUFAs are abundant in most crop seeds and vegetable oils (i.e. canola, soybean, corn and sunflower) [8, 10].

Epigenetic modifications of the DNA play an important role in the molecular development of traits. One major epigenetic mark, DNA methylation (DNAm), occurs on the 5th carbon of cytosines, forming a 5-methylcytosine, and is mainly found in cytosines followed by guanines [11]. Epigenome-wide association studies (EWAS) investigate and identify the common variation in the DNA methylome using genome-wide technology [12, 13].

Many studies have shown that the effect of PUFAs on non-communicable chronic diseases might be mediated by DNAm [14]. For n-3 PUFA, Tremblay and collaborators found methylation changes following eicosapentaenoic acid (EPA) and docosahexaenoic acid (DHA) intake in genes related to inflammatory and immune responses, lipid metabolism, cardiovascular signaling and type 2 diabetes in 36 overweight and obese individuals[15], while Amaral and collaborators found changes related to lipid metabolism, inflammatory response and phagocytosis in 12 overweight and obese young women[16]. n-6 PUFA intake was found to be associated with methylation in the tumor necrosis factor- α (TNF-α) pathway and inflammation processes in a group of 40 normal-weight young women [17]. Despite the evidence relating PUFA intake and DNAm, none of the studies have been conducted in a population-based setting, relying on small sample sizes instead. Our aim was to conduct an EWAS to identify the impact of PUFA intake on DNA methylation using the Cooperative Health Research in the Region of Augsburg (KORA) FF4 cohort and the Leiden Longevity Study (LLS).

## Results

### Samples characteristics

Characteristics of the study populations are given in Table 1. In the KORA FF4 study, the percentage of females (53%) was slightly larger than males (47%) and the mean age was 58.76 years old. Similar characteristics were found in the LLS study whereby a higher percentage of the population were female (52.8%) and the mean age was 58.84 years old. With regards to PUFA measures in the KORA study, alpha-linolenic acid (ALA) was the most daily consumed PUFA n-3 (1069.88 mg/day) followed by docosahexaenoic acid (DHA), eicosapentaenoic acid (EPA), docosapentaenoic acid (DPA) and stearidonic acid (SDA). The most consumed PUFA n-6 was linoleic acid (LA, 8259.37 mg/day), followed by arachidonic acid (ARA) and eicosadienoic acid (EDA). Of the eight PUFA measured in KORA, only three PUFA n-3 and one PUFA n-6 (LA) were available in LLS; in this cohort, ALA was also the PUFA n-3 with the highest daily consumption, followed by DHA and EPA.

**Table 1:** Characteristics the KORA FF4 and LLS study participants

Characteristic	KORA FF4 (*n* = 1354)	LLS (*n* = 488)
Age (years)	58.8 (11.3)	58.8 (6.3)
Female	718 (53.0%)	258 (52.9%)
BMI (kg/m2)	27.6 (4.99)	25.3 (3.4)
*Smoking*		
Current	200 (14.8%)	61 (12.5%)
Former	587 (43.4%)	275 (56.4%)
Never	567 (41.9%)	152 (31.1%)
*Physical activity*		
Active	827 (61.1%)	–
Non-active	527 (38.9%)	–
Energy intake (kcal)	1851.9 (405.1)	1992.8 (602.0)
*Current estrogen therapy*		
Yes	42 (5,8%)	–
No	676 (94.2%)	–
*PUFA supplement intake*		
Yes	33 (2.4%)	–
No	1321 (97.6%)	–
*PUFA n-3 intake (mg/day)*		
ALA	1069.88 (311.81)	1045.30 (622.18)
SDA	41.99 (27.25)	–
EPA	147.06 (82.40)	77.32 (68.19)
DPA	56.43 (16.31)	–
DHA	203.31 (86.78)	125.92 (108.10)
*PUFA n-6 intake (mg/day*)		
LA	8259.37 (2232.79)	13,307.00 (6530.12)
EDA	46.61 (21.24)	–
ARA	195.34 (56.33)	–

Data are mean (SD) or n (%). BMI: Body mass index, PUFA: Polyunsaturated fatty acid, ALA: Alpha-linolenic acid, SDA: Stearidonic acid, EPA: Eicosapentaenoic acid, DPA: Docosapentaenoic acid, DHA: Docosahexaenoic acid, LA: Linoleic acid, EDA: Eicosadienoic acid, ARA: Arachidonic acid

### Correlation between different types of PUFA intake

We ran Pearson correlation analyses between all the PUFAs from the KORA study (Additional file 2: Supplementary Table 1). We found a strong positive correlation coefficient between ALA and LA (*r* = 0.76), SDA and EPA (*r* = 0.99), SDA and DHA (*r* = 0.97), DPA and EPA (*r* = 0.79), DHA and EPA (*r* = 0.98), DPA and DHA (*r* = 0.85) and EDA and ARA (*r* = 0.86).

### Epigenome-wide association analyses in KORA FF4

**Fig. 1:**
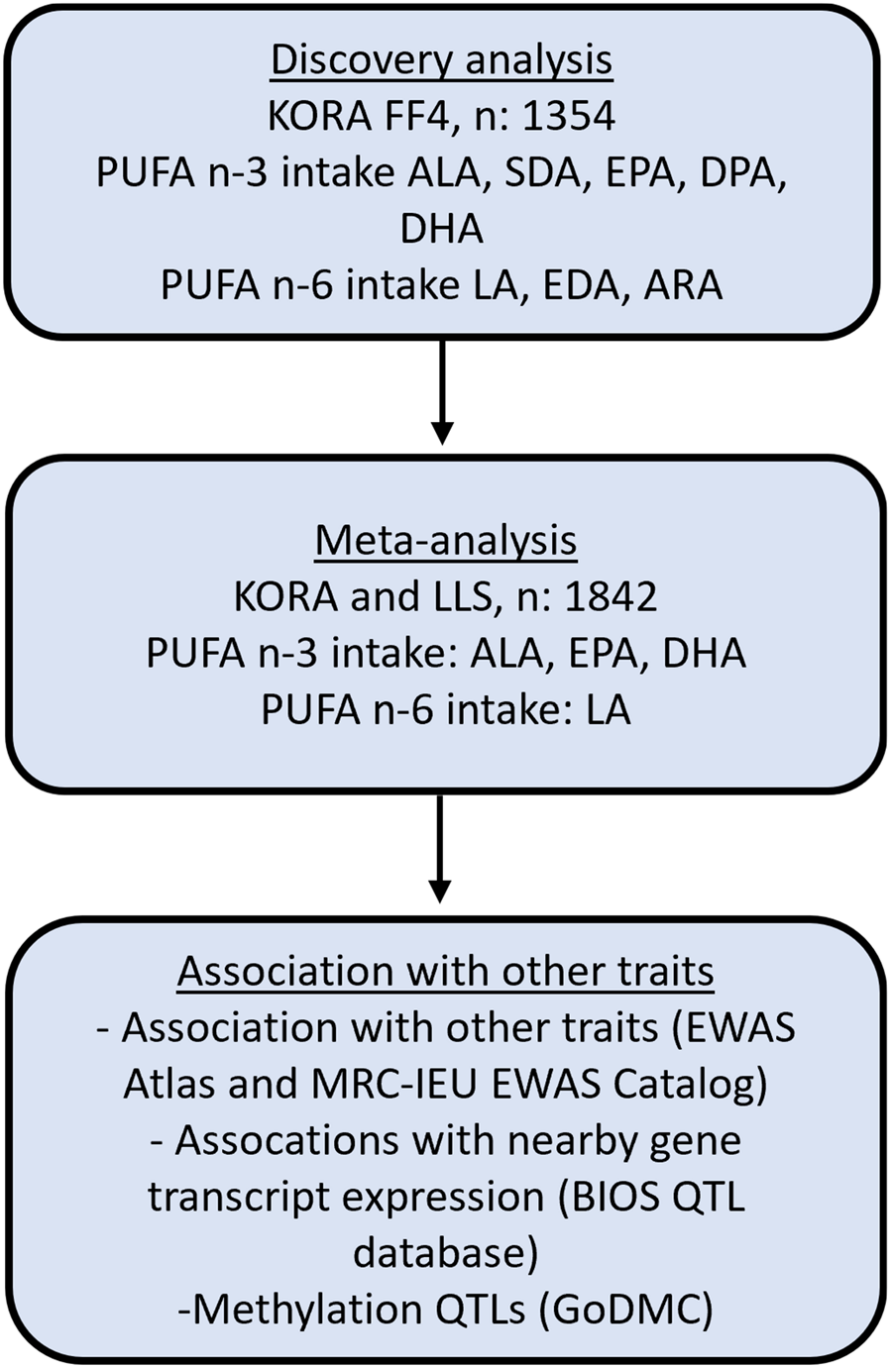
EWAS Workflow: assessing PUFA Intake's Impact on DNA Methylation Using KORA FF4 and LLS Cohorts. KORA: Cooperative Health Research in the Region of Augsburg, PUFA: Polyunsaturated fatty acid, ALA: Alpha-linolenic acid, SDA: Stearidonic acid, EPA: Eicosapentaenoic acid, DPA: Docosapentaenoic acid, DHA: Docosahexaenoic acid, LA: Linoleic acid, EDA: Eicosadienoic acid, ARA: Arachidonic acid, LLS: Leiden Longevity Study

We identified two differentially methylated positions (DMPs) associated with PUFA intake (Table 2) in the KORA study. A workflow diagram illustrating the study design is shown in Fig. 1. For the DMP cg05041783, annotated to the gene *MARK2*, we found a 0.01% increase in DNA methylation per mg/day increase of DPA (fully adjusted beta: 9.81 × 10^–5^, 95% CI: 6.25 × 10^–5^-1.33 × 10^–4^, *P* value: 6.75 × 10^–8^) (Additional file 1: Suppl. Figure 1), while DMP cg19937480, annotated to the gene *PRDX1*, was positively associated with a per mg/day intake of DHA in model 1 only (beta: 2.0 × 10^–5^, 95% CI: 1.28 × 10^–5^-2.73 × 10^–5^, *P* value: 6.98 × 10^–8^) (Additional file 1: Suppl. Figure 2). The top 20 DMPs for the fatty acids SDA, DPA, EDA and ARA which were analyzed in the KORA study only are shown in Additional file 2: Supplementary Table 2.

**Table 2:** EWAS between genome-wide DNA methylation and PUFA intake in the KORA study

					Model 1						Model 2					
PUFA	CpG	CHR	Position	Gene	Beta	SE	Lower 95% CI	Upper 95% CI	p value	N	Beta	SE	Lower 95% CI	Upper 95% CI	p value	N
DHA	cg19937480	1	45,987,798	*PRDX1*	2.00E-05	3.69E-06	1.28E-05	2.73E-05	6.98E-08*	1349	1.92E-05	3.95E-06	1.15E-05	2.69E-05	1.24E-06	1349
DPA	cg05041783	11	63,656,223	*MARK2*	8.43E-05	1.60E-05	5.29E-05	1.16E-04	1.65E-07	1337	9.8E-05	1.81E-05	6.25E-05	1.33E-04	6.75E-08*	1337

EWAS: Epigenome-wide association study, PUFA, Polyunsaturated fatty acid, CHR: Chromosome, SE: Standard Error, 95% CI: 95% Confidence Interval, DHA: Docosahexaenoic acid, DPA: Docosapentaenoic acid. Model 1: adjusted for age, sex, BMI, smoking, WBC% and technical variables. Model 2: same as Model 1 further adjusted for physical activity, energy intake, estrogen therapy and supplement intake (if applicable). *statistically significant (Bonferroni threshold 7.17 × 10^–8^)

### Meta-analysis results

**Fig. 2:**
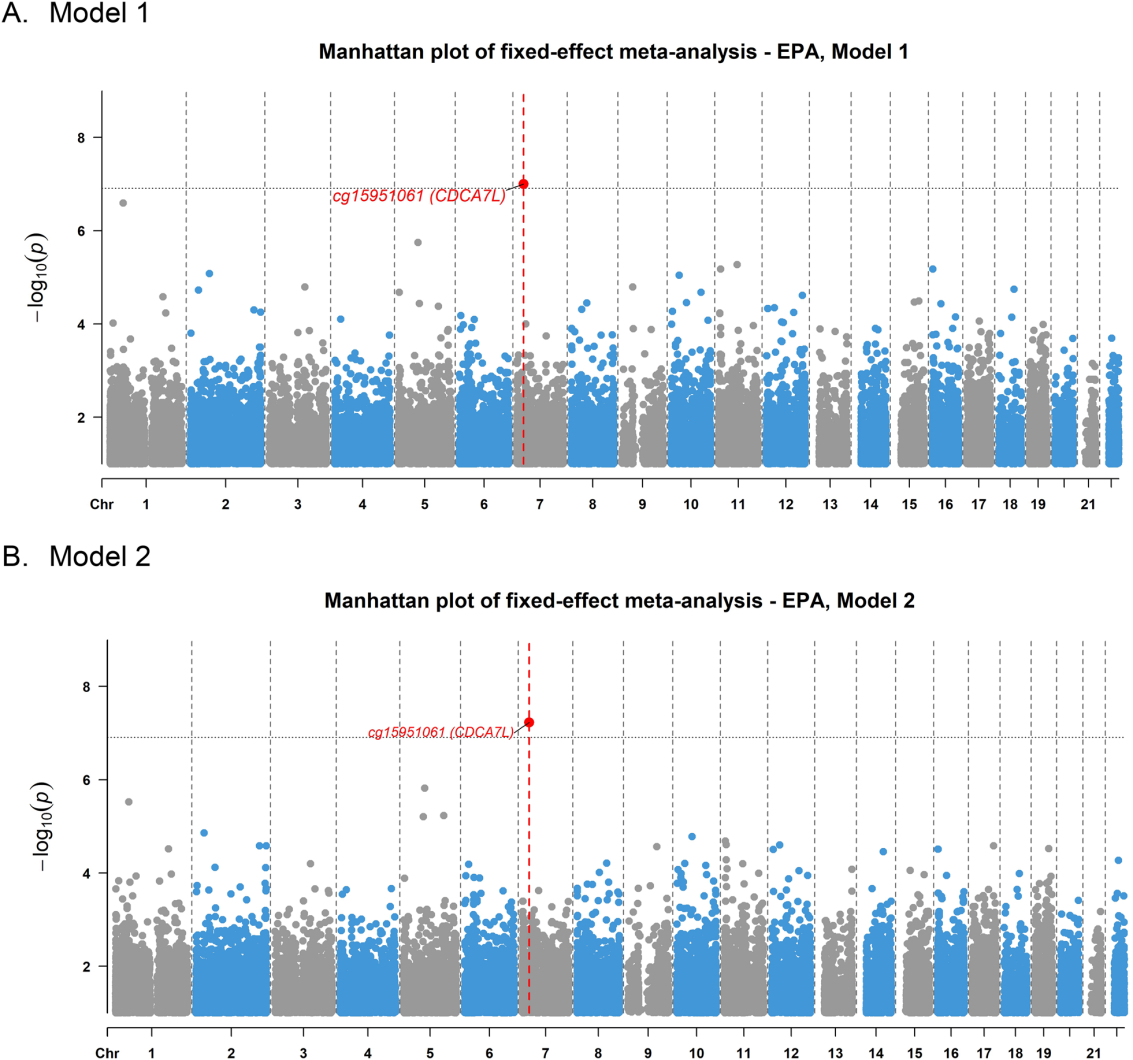
Manhattan plot of meta-EWAS results of EPA Panel **A**. Manhattan plot of results from meta-analysis of epigenome-wide association studies on EPA using linear regression models adjusting for age, sex, BMI, smoking, WBC% and technical variables. The x-axis shows the chromosomal position, and the y-axis the -log10 p value of the DMP-PUFA association. The horizontal gray line indicates the genome-wide significance threshold at a Bonferroni-corrected p value lower than 0.05 (alpha = 1.23 × 10 − 7). The red dot represents the significant DMP identified in this analysis labeled with the cpg name and its annotated gene name. Panel **B**. Manhattan plot of results from meta-analysis of epigenome-wide association studies on EPA using linear regression models adjusted for age, sex, BMI, smoking, WBC%, technical variables, physical activity, energy intake, estrogen therapy and PUFA supplement intake

The association analyses could be extended for four PUFAs (ALA, DHA, EPA and LA) by conducting a meta-analysis of EWAS in KORA and LLS, where two significant DMPs were identified (Table 3). Cg15951061, annotated to the gene *CDCA7L,* was associated with EPA intake (effect of 1 mg/day increased intake on fully adjusted beta: 2.19 × 10^–5^, 95% CI: 1.41 × 10^–5^—2.97 × 10^–5^, *P* value = 5.91 × 10^–8^). This positive association was significant in both models (Fig. 2, Additional file 1: Suppl. Figure 3) and its direction of effect consistent in KORA and LLS. Cg19937480 (*PRDX1*), the same CpG identified in the analysis in KORA alone, was associated with DHA intake in model 1 (1 mg/day increased intake beta: 2.00 × 10^–5^, 95% CI: 1.27 × 10^–5^-2.73 × 10^–5^, *P* value = 6.00 × 10^–8^; Additional file 1: Suppl. Figure 4); this association was nominally significant in the fully adjusted model (Additional file 1: Suppl. Figure 5), but the directions of effect were opposite in KORA and LLS. The top 20 DMPs for ALA, DHA, EPA and LA are shown in Additional file 2: Supplementary Table 3.

**Table 3:** EWAS meta-analysis results between genome-wide DNA methylation and PUFA intake in KORA and LLS

					Model 1						Model 2					
PUFA	CpG	CHR	Position	Gene	Beta	SE	Direction	95% CI	p value	N	Beta	SE	Direction	95% CI	p value	N
DHA	cg19937480	1	45,987,798	*PRDX1*	2.00E-05	3.70E-06	+ −	1.27E-05 − 2.73E-05	5.80E-08*	1834	1.92E-05	3.90E-06	+−	1.16E-05 − 2.68E-05	1.19E-06	1834
EPA	cg15951061	7	21,946,025	*CDCA7L*	2.07E-05	3.9E-06	+ +	1.31E-05 - 2.83E-05	1.00E-07*	1834	2.19E-05	4.00E-06	+ +	1.41E-05 − 2.97E-05	5.91E-08*	1834

EWAS: Epigenome-wide association study, PUFA, Polyunsaturated fatty acid, CHR: Chromosome, SE: Standard Error, Direction: direction of effect in the individual cohorts, KORA and LLS, 95% CI: 95% Confidence Interval, DHA: Docosahexaenoic acid, EPA: Eicosapentaenoic acid. Model 1: adjusted for age, sex, BMI, smoking, WBC% and technical variables. Model 2: same as Model 1 further adjusted for physical activity, energy intake, estrogen therapy and supplement intake (if applicable). *statistically significant (Bonferroni threshold 1.23 × 10^–7^)

### Limited evidence for association with other traits

To understand possible mechanisms between CpG site methylation and PUFA-related health outcomes, we searched for associations between the 3 CpG sites (cg19937480, cg05041783, cg15951061) and other traits using the EWAS Atlas and the MRC-IEU EWAS Catalog [18, 19]. The DMP cg19937480 was found to be linked to rheumatoid arthritis and both cg19937480 and cg15951061 to aging factors; however, for cg05041783, we found no associations. We also searched the BIOS QTL database [20] to identify whether the CpG sites were associated with nearby gene transcript expression, but found no associations. We also attempted to search for any methylation quantitative trait loci (mQTLs) using GoDMC [21] to carry out further causal analyses, but also found no SNPs associated with methylation of these DMPs.

## Discussion

In this study, we explored whether the consumption of n-3 and n-6 PUFAs might be associated with DNA methylation of leukocytes, suggesting a possible pathway in which PUFA intake influences health outcomes at the molecular level.

We ran Pearson’s correlation analyses between the PUFAs analyzed in this study, where the strongest correlation coefficients were found between PUFAs that come mainly from the same diet source. EPA, SDA, DHA and DPA are mainly found in marine sources in different quantities [22, 23]; ARA and EDA are found in meat sources [24].

We identified two DMPs associated with two PUFAs (DHA and DPA) in the KORA FF4 population alone. The first DMP (cg05041783), located in the gene *microtubule affinity regulating kinase 2* (*MARK2),* was found to be associated with DPA intake. This DMP has not been described in the literature. The gene *MARK2* encodes the serine/threonine-protein kinase MARK2, a member of the MARK (microtubule affinity regulating kinase) family that is involved in a great diversity of biological functions, including cell polarity, cell cycle control, cell signaling, protein stability and control of microtubule dynamics [25]. It is suggested that the protein MARK2 is involved in early phosphorylation of tau, a protein that when phosphorylated plays an important role in Alzheimer’s disease [26]. There is evidence that n-3 PUFA intake, including DPA, offers benefits concerning the development of neurological disorders including Alzheimer’s disease [27] and its derived lipid mediators play a key role in inflammatory response having neuroprotective potential, but its mechanisms are not yet elucidated [28]. There is also evidence that the protein MARK2 plays a role in glucose homeostasis by regulating insulin metabolism in mice through Raf/MEK/ERK MAP kinase cascade in insulin-glucose metabolism [29]. However, studies show that n-3 PUFAs may affect insulin resistance and secretion in humans through inflammatory pathways [4]. Of note, the association between cg05041783 and DPA intake could not be studied in LLS, as this PUFA was not available in this cohort.

For four PUFAs also available in LLS, a meta-analysis including both studies was conducted to increase sample size. The KORA study and subsequent meta-analysis identified the DMP cg19937480, located within the *PRDX1* gene, as significantly associated with DHA intake in the model adjusted for age, sex, BMI, smoking, WBC% and technical variables. However, at the single-study level, the direction of effect was inconsistent, suggesting the association observed in the meta-analysis to be driven by the effect observed in KORA. These differences may be explained by largely different sample sizes, heterogeneity and potential inaccuracy in the estimation of dietary intake variables, as well as the small size of the effects presented. Moreover, this association was not significant after further adjustment for other potential confounders (like energy intake), which further suggests the association between this DMP and DHA intake might be confounded. This DMP has been associated not only with rheumatoid arthritis [30], but also with aging processes [31, 32]. The *PRDX1* gene, known for its association with oxidative stress, has been implicated as a tumor suppressor in several cancers [33].

The second DMP, cg15951061, was associated with EPA intake in both meta-analysis models and its positive direction of effect at the single-study level was consistent. This DMP has also previously been described to be associated with aging [31]. Its annotated gene, *CDCA7L*, further supports this association with its links to both rheumatoid arthritis and aging [30–32]. Given the recognized role of DHA and EPA intake in influencing inflammatory responses and the increased oxidative stress and chronic inflammation that characterize aging, these findings suggest a multifaceted relationship among DHA and EPA intake, methylation patterns, oxidative stress, inflammation, and the aging process. However, further research is required to better understand these relationships.

Some strengths and limitations should be considered in our study to interpret our reported findings. A strength of this study is that it was carried out in a large sample (*N* = 1354) of the general population. Currently, our study is the only population-based study concurrently analyzing genome-wide DNAm data and PUFAs. All similar studies in the literature evaluated smaller samples [14], where the largest one had a sample of 517 pregnant women and assessed the use of DHA supplementation only [33]. The use of the Illumina MethylationEPIC BeadChip microarray was also a strength. The EPIC BeadChip has 413,743 additional CpG sites in comparison with its precursor the Illumina HumanMethylation450 [34], providing an increased genome coverage. Since DNAm is tissue specific, a limitation to our study was the use of whole blood samples. To identify the effects of PUFA intake concerning inflammation and immune response, leukocytes are a relevant tissue. However, to analyze the effect of PUFA metabolism, liver tissues could be more appropriate. The blended approach to dietary assessment used in our study was considered to be more accurate than the same instruments used alone, but validation with biomarkers has not yet been performed [35], and some imprecision due to participant reporting cannot be ignored. Although we carried out the EWAS in a large population, the replication of our exploratory study in larger independent cohorts would be desirable to ensure the reproducibility of our findings. Future research could use targeted DNAm profiling in leukocytes and liver tissue to confirm the methylation status of the genes identified here, as well as conduct comparative work on biomarker-based and FFQ-based PUFA measures in relation to DNAm.

## Conclusions

Our study is the first to report an association between 2 CpG sites (cg19937480, cg05041783) with PUFA intake (DHA and DPA, respectively) and our meta-analysis revealed a further association between cg15951061 and EPA. However, there appears to be little understanding within the literature as to the mechanisms of these CpG sites and therefore further investigation is needed to elucidate the consequence of CpG site methylation on PUFA-related health outcomes.

## Methods

### Study populations

#### Cooperative health research in the region of Augsburg (KORA)

KORA FF4 (2013–2014) is the second follow-up of KORA S4 cohort. Its baseline was conducted between 1999–2000 with participants of German nationality from the region of Augsburg, Germany, aged between 25 and 74 years old. From the 4261 participants enrolled in KORA S4, 2279 also participated in the second follow-up study. The KORA cohort ethical approval was granted by the ethics committee of the Bavarian Medical Association (REC reference numbers FF4: #06068) and it was carried out in accordance with the principles of the Declaration of Helsinki. Details about the KORA FF4 study protocol and recruitment were published elsewhere [36].

From the 2279 participants in the KORA FF4 study, individuals without DNAm or dietary data, pregnant women, and participants with severe blood disorders were excluded, resulting in a total number of 1354 participants.

#### Leiden longevity study (LLS)

The Leiden Longevity Study (LLS) [37] was established in 2002, with the aim of investigating the genetic component of exceptional survival and its interaction with environmental factors. From 2002 to 2006, long-lived, Dutch, Caucasian siblings (*n* = 944) were recruited with their offspring (*n* = 1671) and their offspring’s partners (*n* = 744).

Family eligibility required at least two long-lived living siblings who met a stringent, sex-specific age criterion (aged at least 89 years for males and 91 years for females). At the time of the study’s initiation, less than 0.5% of the Dutch population fulfilled this requirement as an individual, and sibships with multiple eligible members were estimated to represent less than 0.1% [38].

In the LLS population, offspring and other first-degree relatives are enriched for familial influences on longevity [37]. Their partners serve as controls, having comparable age, socio-economic status, location, lifestyle and environmental factors but without this genetic advantage. Recruited, living subjects completed a pedigree, questionnaires, and a non-fasted venous blood sample was drawn for isolation of DNA, RNA, serum, and plasma.

### Dietary polyunsaturated fatty acid intake

#### Cooperative health research in the region of Augsburg (KORA)

The participants were requested to answer at least two 24-h food lists (24HFL) and a food frequency questionnaire (FFQ) [35, 39]. Through a two-step model, the daily food intakes were estimated. In step one, a logistic linear mixed model was applied to estimate the probability of food item consumption for each participant based on the 24HFL data. The models were adjusted for age, sex, BMI, physical activity, smoking, education, and additionally for the frequency of food consumption (assessed by the FFQ). Since the 24HFL does not assess the amounts of food consumed, these were estimated in step two using data from the second Bavarian food consumption survey (BVS II), a cross-sectional study to assess the dietary habits of the Bavarian population. The amount distribution was not symmetrical, so the quantities were transformed using Box-Cox transformation and modeled using linear mixed-effect models adjusted for age, sex, BMI, smoking, physical activity and educational level. The estimated quantities were then transformed back to the original scale, obtaining the amount consumed per individual per item. By multiplying the probability of consumption and the estimated amount, the individual's usual intake of each food item was estimated. The nutrients provided by each food item were taken from the German Food Composition Table Bundeslebensmittelschlüssel (Version 3.0.2) [35].

The usual intakes (in mg per day) of the n-3 PUFAs alpha-linolenic acid (ALA, (C18:3, n-3)), stearidonic acid (SDA, (C18:4, n-3)), eicosapentaenoic acid (EPA, (C20:5, n-3)), docosapentaenoic acid (DPA, (C22:5, n-3)) and docosahexaenoic acid (DHA, (C22:6, n-3)), and the n-6 PUFAs linoleic acid (LA, (C18:2, n-6)), eicosadienoic acid (EDA, (C20:2, n-6)) and arachidonic acid (ARA, (C20:4, n-6)) were estimated for each participant and used as exposure variables in the EWAS models.

#### Leiden longevity study (LLS)

In the LLS study, a self-administered FFQ was used to assess dietary intake. The daily intake of the four fatty acids ALA, EPA, DHA and LA was estimated. The FFQ was designed for the Dutch population and based on the Vet Express [40] and extended with vegetables, fruit and foods to 104 items for estimating the intake of specific PUFA’s and other nutrients. The participants were asked to report the food intake during the previous month [41].

### White blood cells percentage

The percentage of monocytes, basophils, eosinophils, neutrophils and lymphocytes in whole blood from each participant from the KORA study analysis was determined through differential blood count from the participant’s blood sample using the Coulter LH 750 device from Beckman Coulter and the Sysmex XN device. Three participants with malignant neoplasm of lymphatic and hematopoietic tissue (ICD9 codes: 200–208) were excluded from our analysis.

In the LLS, the percentage of white blood cell (WBC) types (neutrophils, lymphocytes, monocytes, eosinophils, and basophils) was measured with a blood Differential test in fasted blood samples. These cell types were included in all analyses.

### DNA methylation data

#### Cooperative health research in the region of Augsburg (KORA)

Genomic DNA extracted from whole blood from 1928 individuals from KORA FF4 was bisulfite converted using the EZ-96 DNA Methylation Kit (Zymo Research, Orange, CA, USA) in two batches (*N* = 488, *N* = 1440). Subsequent methylation analysis was performed on an Illumina (San Diego, CA, USA) iScan platform using the Infinium MethylationEPIC BeadChip according to standard protocols provided by Illumina.

Raw DNA methylation data were extracted with Illumina Genome Studio (version 2011.1), methylation module (v1.9.0), and processed using R (v3.0.1) following the CPACOR pipeline of Lehne et al. [42] including exclusion of 65 SNP probes and background correction using minfi [43]. Probes were set to missing if the detection *p* value ≥ 0.01 or the number of beads < 3. Samples were excluded if the detection rate was ≤ 0.95. Quantile normalization was performed on intensity values separated by color channel, probe type and M/U subtypes. The resulting methylated and unmethylated signals were used to calculate β values, a measure of percent methylation between 0 and 1.

#### Leiden longevity study (LLS)

DNA methylation data of whole blood samples were generated from 821 unrelated LLS participants by the Human Genotyping facility (HuGe-F, Erasmus MC, Rotterdam, The Netherlands) within the Biobank-Based Integrative Omics Studies (BIOS) consortium. DNA of LLS was analyzed using the Illumina 450 k BeadChip array. Genomic DNA (500 ng) was isolated and bisulfite converted using the Zymo EZ-96 DNA methylation kit (Zymo Research Corp, Irvine, CA, USA). 4 μl was then hybridized on the Infinium HumanMethylation450 BeadChip array (Illumina Inc, San Diego, CA, USA) according to the manufacturer’s protocol.

IDAT files were generated by the Illumina iScan BeadChip scanner and data quality was assessed in R using sample dependent and sample independent quality metrics reported by the Bioconductor package MethylAid (van Iterson et al., 2014) with default settings. Unreliable or outlying values were removed, including those indistinguishable from background noise (detection *p* value > 0.01), based on a low number of beads (*n* < 3), or with zero values for signal intensity. Following background correction and probe-type normalization, the data were checked for outlying samples using plots of the first two principal components (PCs) and any samples or probes with less than 95% success rate were removed. However, there were no outliers found in these checks indicating high quality data. The resulting methylated and unmethylated signals were used to calculate β values which range from 0 (completely unmethylated state) to 1 (completely methylated).

### Potential confounding variables

The covariates selected for this study were age (years), sex (male/female), BMI (kg/m^2^), smoking (current/former/never smoker), WBC%, physical activity (active/ non-active), energy intake (kcal/day), current estrogen therapy (yes/no) and PUFA supplement intake (yes/no).

Physical activity was assessed on a four-level graded scale based on the amount of regular leisure time exercise per week during summer and winter in KORA. Based on this assessment, participants were categorized into active and non-active [44].

In LLS, a base model was run adjusting for age (years), sex (Male/female), BMI (kg/m2), smoking status (current/former/never), blood cell type proportions (monocytes, basophils, eosinophils, neutrophils, lymphocytes), and plate number.

### Statistical analysis

In KORA, the association analysis between DNAm beta values and each PUFA was carried out in R, using linear regression model, with the methylation beta value as the dependent variable and the PUFA intake as the independent variable. To adjust for potential confounding, surrogate variables were calculated for each PUFA using the sva R package [45]. The model was adjusted for age, sex, BMI, smoking, WBC% and surrogate variables (Model 1). WBC% (monocytes, basophils, eosinophils, neutrophils and lymphocytes) were calculated from blood subfractions. We removed probes found on sex chromosomes and those that contain common genetic variants. We also removed probes that were ambiguously mapped as well as probes that were removed from the Illumina arrays [46]. The total number of probes was 697,732 and therefore we used a Bonferroni threshold of 7.17 × 10^–8^ (0.05/697,732) to determine significance.

In LLS, the association analysis between DNAm beta values and each PUFA was carried out in R package limma, with the methylation beta value as the dependent variable and PUFA intake as the independent variable. The base model was adjusted for age, sex, BMI, smoking, WBC% and plate number (Model 1) all as fixed effects.

We ran a second model (model 2) similar to the main one (model 1) but further adjusted for physical activity, energy intake, estrogen therapy and, if applicable, PUFA supplement intake, added to the model as fixed effects. The PUFA supplement intake was used as confounder only for the models analyzing ALA, DHA and EPA as independent variable. In LLS, only total energy intake (kcal/day) was additionally adjusted for in the extended model. We performed quality control for our analysis and the Manhattan and QQ plots can be found in the Supplementary Materials.

### Meta-analysis

For the meta-analysis, we performed inverse variance fixed effects meta-analysis using METAL version 2011–03-25 [47]. For multiple testing correction, we used Bonferroni correction. A p value < 1.23 × 10^–7^ (0.05/406,132 DMPs) was used as the significance threshold.

### Supplementary Information

**Additional file 1**. Supplementary information.

**Additional file 2**. Supplementary tables.

## Data Availability

The informed consents given by KORA study participants do not cover data posting in public databases. However, data are available upon request from KORA Project Application Self-Service Tool (https://epi.helmholtz-muenchen.de/). Data requests can be submitted online and are subject to approval by the KORA Board.

## Abbreviations

ALA: Alpha-linolenic acid (C18:3, n-3)
ARA: Arachidonic acid (C20:4, n-6)
BVS II: Bavarian food consumption survey
BIOS: Biobank-Based Integrative Omics Studies
DMPs: Differentially methylated positions
DNAm: DNA methylation
DHA: Docosahexaenoic acid (C22:6, n-3)
DPA: Docosapentaenoic acid (C22:5, n-3)
EDA: Eicosadienoic acid (C20:2, n-6)
EPA: Eicosapentaenoic acid (C20:5, n-3)
EWAS: Epigenome-wide association studies
EPIC: European Prospective Investigation into Cancer and Nutrition
FFQ: Food frequency questionnaire
KORA: Cooperative Health Research in the Region of Augsburg
LLS: Leiden Longevity Study
LA: Linoleic acid (C18:2, n-6)
mQTLs: Methylation quantitative trait loci (mQTLs)
MARK2: Microtubule affinity regulating kinase 2 (MARK2)
MSM: Multiple Source Method
NCI: National Cancer Institute
PUFA: Polyunsaturated fatty acids
PCs: Principal components
24HFL: Repeated 24-h food lists
SDA: Stearidonic acid (C18:4, n-3)
TNF-α: Tumor necrosis factor- α
WBC: White blood cell
