# Supplementary material for: Epigenome-wide association study of dietary fatty acid intake
Source: Clin Epigenetics. 2024 Feb 16;16:29. doi: 10.1186/s13148-024-01643-9 (PMC10874013; doi:10.1186/s13148-024-01643-9)
Supplement: Supplementary file 1 — Additional file 1. Supplementary information. [file 13148_2024_1643_MOESM1_ESM.docx]

# Supplementary information

## Suppl. Figure 1

1. Manhattan plot of EWAS results from DPA in KORA (Model 2)


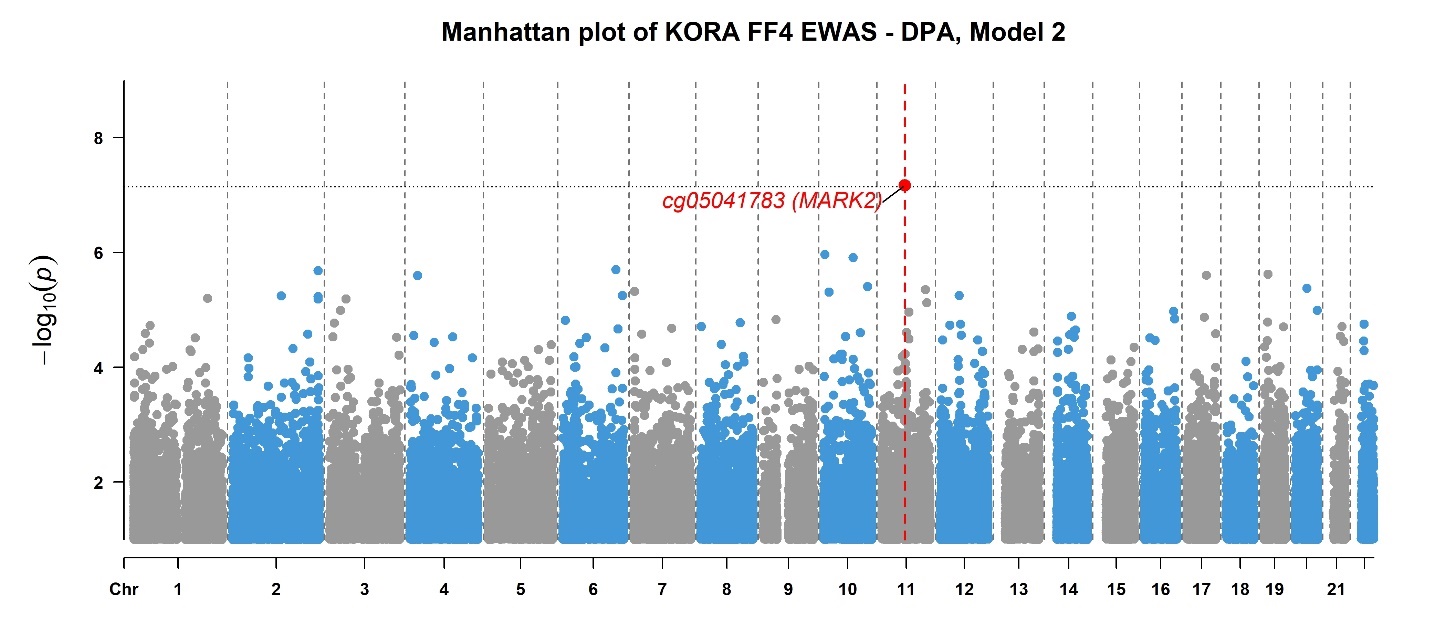


1. QQpplot


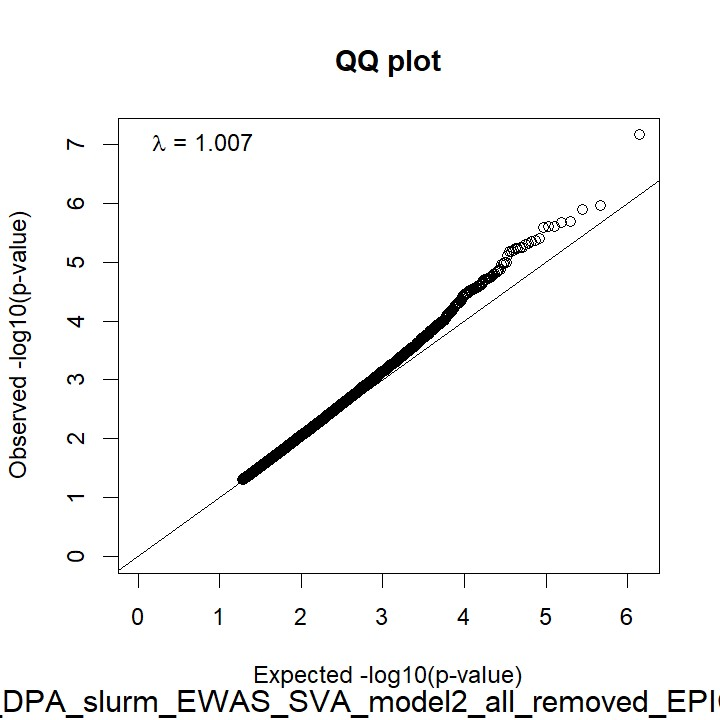


**Panel A.** Manhattan plot of results from epigenome-wide association studies on docosapentaenoic acid (DPA) in KORA using linear regression models adjusting for age, sex, BMI, smoking, WBC%, technical variables, physical activity, energy intake, estrogen therapy and surrogate variables. The x-axis shows the chromosomal position, and the y-axis the -log10 pvalue of the CpG-PUFA association. The horizontal grey line indicates the genome-wide significance threshold at a Bonferroni-corrected p value lower than 0.05 (alpha = 7.7 × 10 −8). The red dot represents the significant DMP identified in this analysis labeled with the cpg name and its annotated gene name. **Panel B.** Quantile-quantile (QQ) plot to verify over/undersignificance in the results, and lambda value representing the inflation of p-values compared to a normal distribution of p-values.

## Suppl. Figure 2

1. Manhattan plot of EWAS results from DHA in KORA (Model 1)


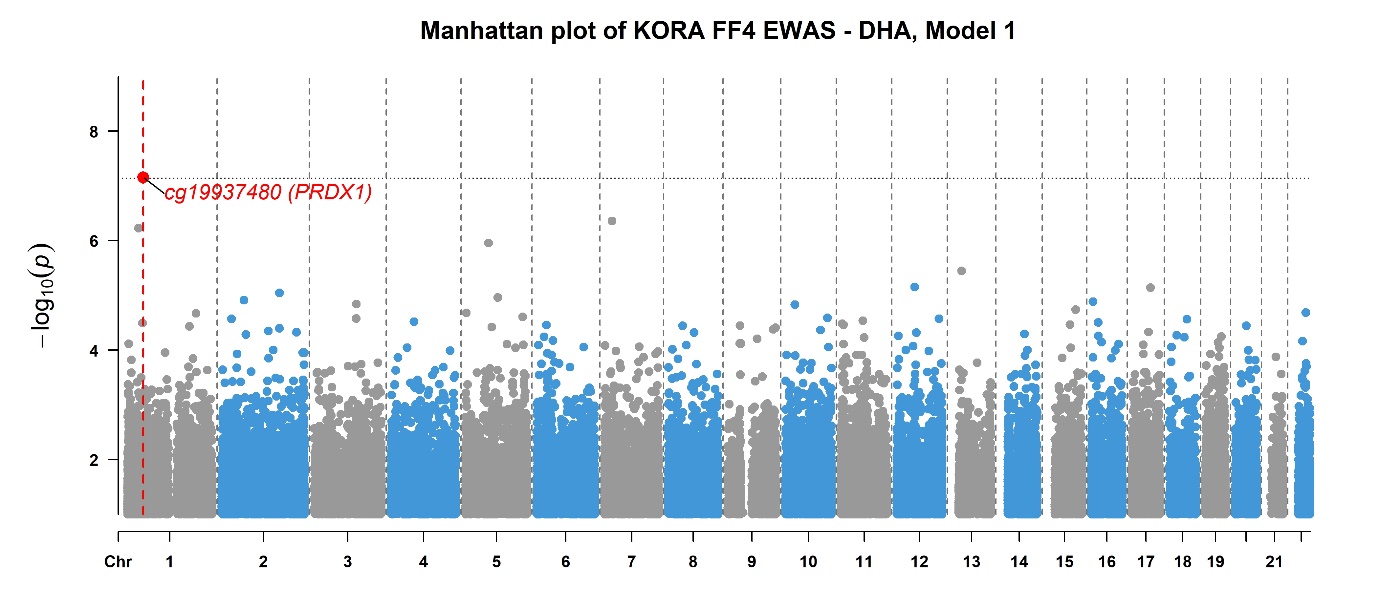


1. QQplot


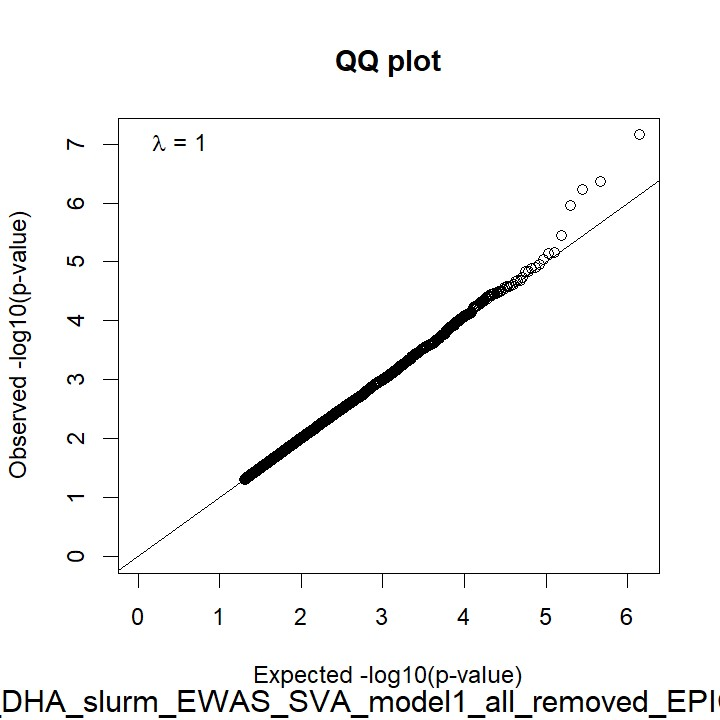


**Panel A.** Manhattan plot of results from epigenome-wide association studies on docosahexaenoic acid (DHA) in KORA using linear regression models adjusting for age, sex, BMI, smoking, WBC%, technical variables and surrogate variables. The x-axis shows the chromosomal position, and the y-axis the -log10 pvalue of the CpG-PUFA association. The horizontal grey line indicates the genome-wide significance threshold at a Bonferroni-corrected p value lower than 0.05 (alpha = 7.7 × 10 −8). The red dot represents the significant DMP identified in this analysis labeled with the cpg name and its annotated gene name. **Panel B.** Quantile-quantile (QQ) plot to verify over/undersignificance in the results, and lambda value representing the inflation of p-values compared to a normal distribution of p-values.

## Suppl. Figure 3

1. QQplot of meta-EWAS results from EPA in KORA+LLS (Model 1)


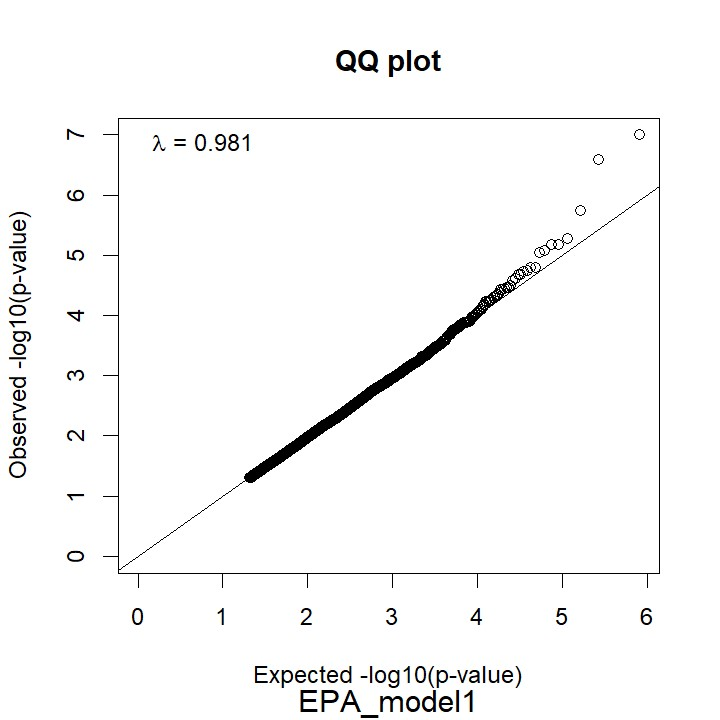


1. QQplot of meta-results from EPA in KORA+LLS (Model 2)


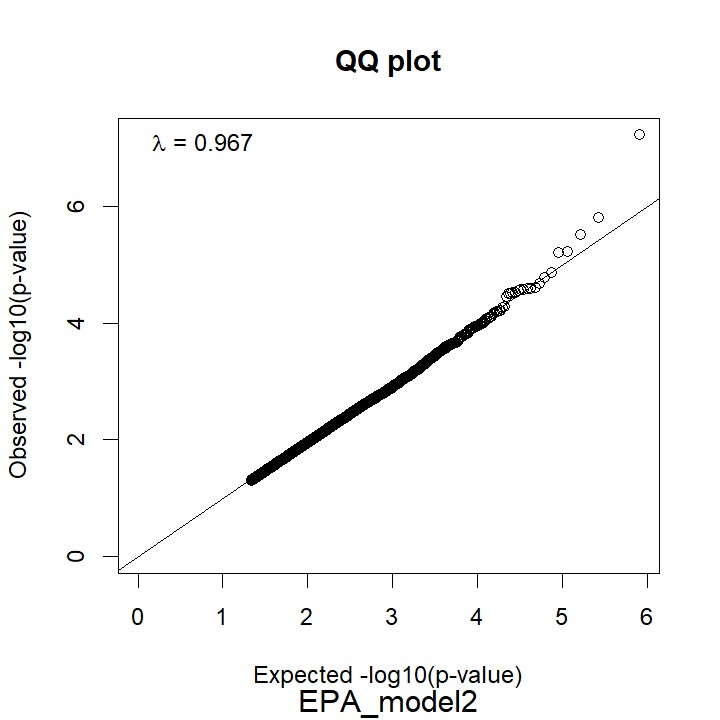


**Panel A and B.** Quantile-quantile (QQ) plots to verify over/undersignificance in the results, and lambda value representing the inflation of p-values compared to a normal distribution of p-values.

## Suppl. Figure 4

1. Manhattan plot of meta-EWAS results from DHA in KORA+LLS (Model 1)


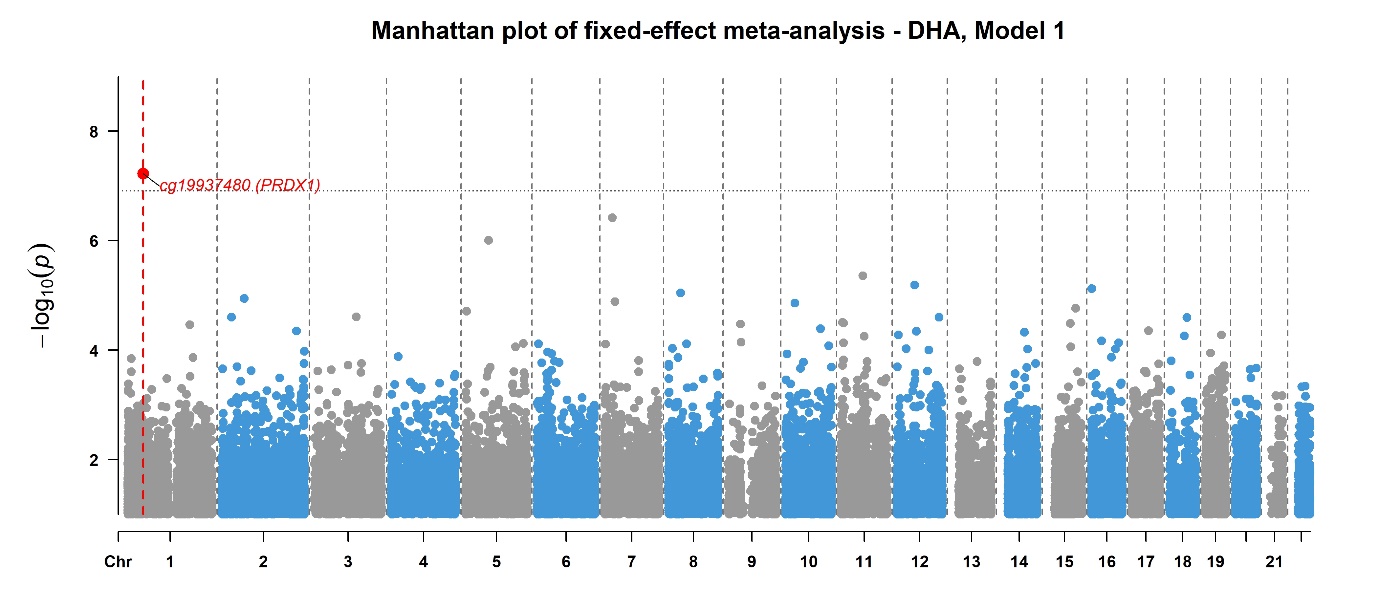


1. QQplot


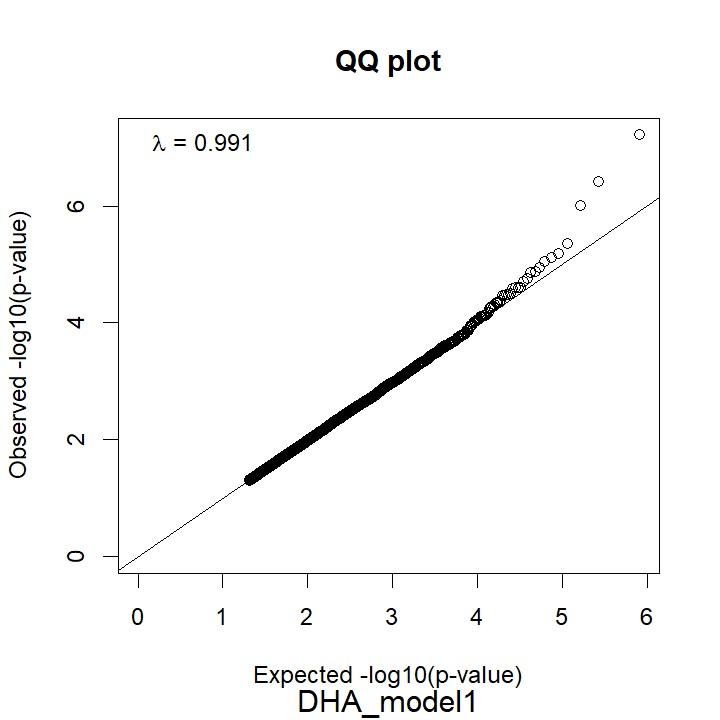


**Panel A.** Manhattan plot of results from the meta-analysis of the epigenome-wide association studies on docosahexaenoic acid (DHA) in KORA and LLS using linear regression models adjusting for age, sex, BMI, smoking, WBC% and technical variables. The x-axis shows the chromosomal position, and the y-axis the -log10 pvalue of the CpG-PUFA association. The horizontal grey line indicates the genome-wide significance threshold at a Bonferroni-corrected p value lower than 0.05 (alpha = 1.23 × 10 −7). The red dot represents the significant DMP identified in this analysis labeled with the cpg name and its annotated gene name. **Panel B.** Quantile-quantile (QQ) plot to verify over/undersignificance in the results, and lambda value representing the inflation of p-values compared to a normal distribution of p-values.

## Suppl. Figure 5

1. Manhattan plot of meta-EWAS results from DHA in KORA+LLS (Model 2)


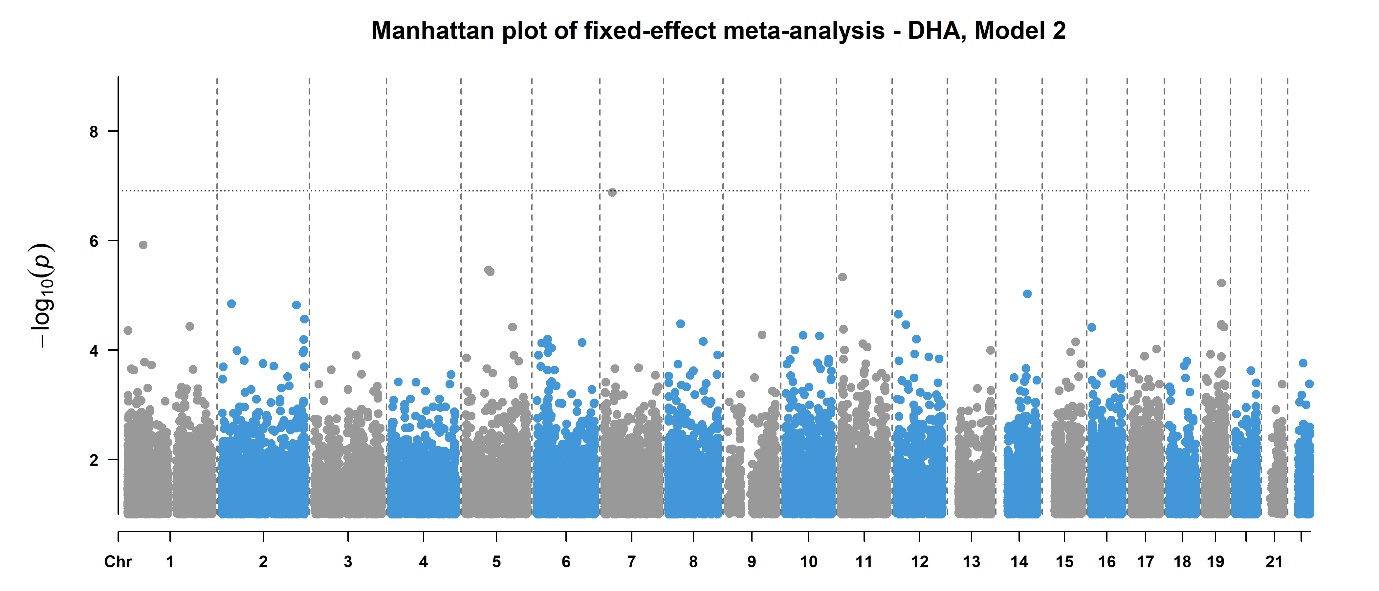


1. QQplot


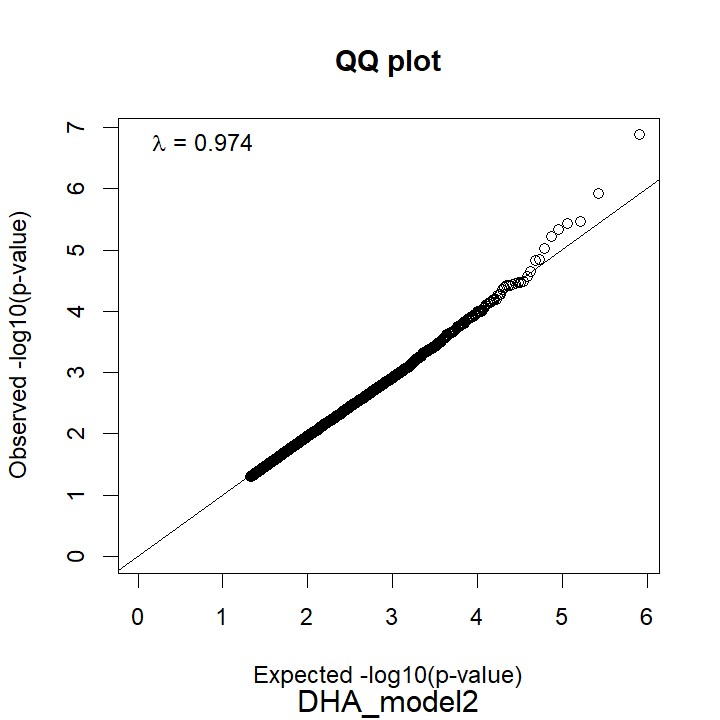


**Panel A.** Manhattan plot of results from the meta-analysis of the epigenome-wide association studies on docosahexaenoic acid (DHA) in KORA and LLS using linear regression models adjusting for age, sex, BMI, smoking, WBC%, technical variables, physical activity, energy intake, estrogen therapy and PUFA supplement intake. The x-axis shows the chromosomal position, and the y-axis the -log10 pvalue of the CpG-PUFA association. The horizontal grey line indicates the genome-wide significance threshold at a Bonferroni-corrected p value lower than 0.05 (alpha = 1.23 × 10 −7). The red dot represents the significant DMP identified in this analysis labeled with the cpg name and its annotated gene name. **Panel B.** Quantile-quantile (QQ) plot to verify over/undersignificance in the results, and lambda value representing the inflation of p-values compared to a normal distribution of p-values.
